# Rising incidence of pancreatic carcinoma in middle-aged and older women-time trends 1961-90 in the city of Malmö, Sweden.

**DOI:** 10.1038/bjc.1996.148

**Published:** 1996-03

**Authors:** M. Hedberg, H. Anderson, A. Borgström, L. Janzon, S. A. Larsson

**Affiliations:** Department of Surgery, Lund University, Malmö General Hospital, Sweden.

## Abstract

The city of Malmö (population 230 000), situated in the south of Sweden, is in an area which has the highest incidence of pancreatic cancer in the country. The present study was designed to assess time trends of the incidence of pancreatic cancer 1961-90. The 1314 incident cases, 651 men and 663 women, were retrieved from the Regional Tumour Register and the National Cause-of-Death Register. In 75% of cases diagnosis was based on autopsy. Twenty per cent of the these cases were first found at autopsy, being undiagnosed. The average age-standardised incidence was 20.4 per 10(5) person-years for men and 13.7 for women. The incidence was higher for men than for women in all age groups above 44 years. No change in incidence over time was observed for men. In older and middle-aged women there was however a statistically significant increase. The average relative change in women above age 64 was 1.7% per year after age adjustment and in women aged 55-64 years 2.6% per year. We have found no results indicating that this increasing incidence could be caused by detection bias as a result of changing autopsy rates during the study period and hence conclude that the observed increase is explained by a growing number of women being exposed to factors with a potential tumour-promoting or -initiating effect.


British Journal of Cancer (1996) 73, 843-846
© 1996 Stockton Press All rights reserved 0007-0920/96 $12.00

### Rising incidence of pancreatic carcinoma in middle-aged and older women – time trends 1961–90 in the city of Malmö, Sweden

M Hedberg[1], H Anderson[3], A Borgström[1], L Janzon[2] and SA Larsson[1]

*Departments of [1]Surgery and [2]Community Health Sciences, Lund University, Malmö General Hospital, S-214 01 Malmö, Sweden; [3]Regional Tumour Registry, University Hospital, Lund, S-221 85 Lund, Sweden.*

**Summary** The city of Malmö (population 230 000), situated in the south of Sweden, is in an area which has the highest incidence of pancreatic cancer in the country. The present study was designed to assess time trends of the incidence of pancreatic cancer 1961–90. The 1314 incident cases, 651 men and 663 women, were retrieved from the Regional Tumour Register and the National Cause-of-Death Register. In 75% of cases diagnosis was based on autopsy. Twenty per cent of these cases were first found at autopsy, being undiagnosed. The average age-standardised incidence was 20.4 per $10^5$ person–years for men and 13.7 for women. The incidence was higher for men than for women in all age groups above 44 years. No change in incidence over time was observed for men. In older and middle-aged women there was however a statistically significant increase. The average relative change in women above age 64 was 1.7% per year after age adjustment and in women aged 55–64 years 2.6% per year. We have found no results indicating that this increasing incidence could be caused by detection bias as a result of changing autopsy rates during the study period and hence conclude that the observed increase is explained by a growing number of women being exposed to factors with a potential tumour-promoting or -initiating effect.

**Keywords:** incidence; pancreatic carcinoma; women

Geographical variations in the occurrence of disease and changes of incidence over time are observations that indicate that environmental factors and lifestyle habits are involved in the causation of a disease.

Malmö, the third largest city in Sweden with about 230 000 inhabitants, had about a 20–40% higher age-standardised incidence of pancreatic cancer compared with Sweden as a whole between 1980–1990; 20.6 *vs* 14.8 per $10^5$ person–years for men and 13.4 *vs* 11.2 per $10^5$ person–years for women.

In Sweden, all new cancer cases are reported by both the clinician and the pathologist or cytologist to a Regional Tumour Register (RTR), from which the information is forwarded annually to the Swedish National Cancer Register (CR), which was founded 1958. Cancer cases detected at autopsy are reported by the pathologist and these cases are included in the official cancer statistics.

The immediate cause-of-death, as well as the underlying and contributory cause-of-death, is reported by the responsible physician, i.e. the clinician, the pathologist or the general practitioner to the National Cause-of-Death Register. The files of the register, for a given year, are then completed in about 3 years.

All cases of pancreatic cancer in Malmö are treated at one hospital. The autopsy rate in Malmö, up until the late 1980s, was around 70% and above (Berge and Lundberg, 1977; Sternby, 1991). Conditions for case retrieval and validation of diagnosis are therefore excellent. This makes Malmö a city suitable for epidemiological studies.

The aim of this study is to describe time trends in incidence of pancreatic carcinoma in Malmö from 1961, when cancer registration in Sweden was first in full operation, to 1990.

### Materials and methods

Cases were retrieved by cross-linking the National Cause-of-Death Register (CDR) and the Regional Tumour Register for Southern Sweden (RTR). During the period from 1961 to 1990 the CDR held 1240 cases of pancreatic cancer and the RTR 1282 cases, reported from the city of Malmö. The CDR contained 113 cases that were not found in the RTR and likewise, 155 cases were only found in the RTR. Altogether 1127 cases were found in both registers and 1395 in either register.

### *Validation of cases*

The hospital records of 268 cases found in only one of the two registers were all collected and scrutinised. Of the 113 cases found only in CDR, 23 cases were erroneously registered as being of pancreatic origin, 22 were found to be endocrine tumours, leaving 68 pancreatic, non-endocrine tumours.

Of the 155 cases found only in the RTR, four cases could not be retrieved, 20 were erroneously registered as being cancer of pancreatic origin or they were registered before 1961, 12 were of endocrine type and 119 were pancreatic, non-endocrine, tumours.

From the cases shared by both registers, 1127, a subsample of 10% was selected at random for validation of diagnosis. Only two (1.8%) of these selected and evaluated 113 cases were not pancreatic carcinomas.

The diagnosis in patients with carcinoma of pancreatic origin was, in about 75% of cases, based on or confirmed at autopsy examination. In about 20% of cases cytological or histological examinations ante mortem were used to establish the diagnosis. In the remaining 5% diagnosis was based only on clinical findings. Thus in 95% of the cases the diagnosis was confirmed by a pathologist or cytologist. These figures are relatively constant over the period studied, but there are more histological (operatively obtained) specimens in the earlier and more cytology (fine needle aspiration biopsies) in the latter part.

In order to evaluate underreporting from the Department of Pathology 10% of cases randomly-selected from the autopsy files from the time period 1961–87 were searched and scrutinised. Eighty-one of the 84 selected cases of pancreatic cancer had been reported to the RTR, another two were listed as underlying or contributory cause-of-death.

### *Incidence calculations*

From 1961 to 1990, the 1127 cases in both the RTR and the CDR together with the validated cases obtained from either

Correspondence: M Hedberg, Department of Surgery, Malmö General Hospital, S-214 01 Malmö, Sweden
Received 19 December 1994; revised 21 September 1995; accepted 6 October 1995

register were used when calculating the incidence. As a complement the incidence based on not validated RTR cases was also determined for the years 1991–93. Demographic data for the Malmö population were taken from Swedish official statistics. Age-, sex- and calendar year-specific incidences were determined for 5 year age classes.

Age-standardised incidences were calculated per calendar year using the 1970 Swedish population as standard. Moreover, the number of cases and person–years and age-standardised incidences were determined for the age groups 35–54, 55–64 and >64 years of age.

Simple linear regression was used to model age-standardised incidence as a function of calendar time and potential autocorrelation between the residuals was tested by means of the Durbin–Watson test (Durbin and Watson, 1950). As a complement, Poisson regression (McCullagh and Nelder, 1989) was used to model the number of incident cases by time with linear link function and number of person–years as offset variable for each of the three age groups. These models all assume a constant annual incidence change. To facilitate comparisons with other studies, the estimated trends are expressed as percentages of the fitted mid-year (1975) value.

### Results

Of the 268 cases found in only one of the two registers used, it was not possible to confirm the diagnosis in 81 cases (30%). An incorrect diagnosis was made in 77 cases, whereas in the remaining four cases hospital records could not be retrieved.

The average age-standardised incidence for the years 1961–90 was 13.7 per $10^5$ person–years for women and 20.4 for men. The majority, 76.7%, of the cases of pancreatic carcinoma were in the age group of 65 years or older, although some cases were found just less than 40 years of age. The average annual incidence was higher in men, except for one age group. The incidence rose with increasing age, doubling several times between 50 and 70 years of age (Table I).

No significant time trend was found among men, whereas among women there was a significant increase in age standardised incidence (ASI). This increase was found between 55 and 64 years of age, but was most pronounced in women older than 64 years (Table II, Figures 1 and 2). The annual absolute change in ASI (per 100 000 person–years) was 0.48 and 1.11 respectively, in these age groups. The annual relative change was 2.6% for women 55–64 years and 1.7% for women older than 64 years. No autocorrelation between residuals was found. Nor did the result change using linear Poisson regression.

For the years 1991–93, when figures are available only from the RTR, the upward trend among women between 55–64 years of age continued, while a decrease was found in the oldest female group.

During the entire period 20% of the cases were incidental findings at autopsy, i.e. clinically undetected ante mortem. The annual average number of incidentally found cases was five in men and six in women.

### Discussion

The age- and sex-adjusted incidence of pancreatic cancer has for many years been higher in Malmö than in Sweden as a

**Figure 1** Pancreatic carcinoma, age-standardised incidence for men in Malmö, 1961–93. The last 3 years (- - -) are represented by unvalidated cases from the RTR only. ■, 35–54 years; ▲ 55–64 years; ●, >64 years.

**Table I** Pancreatic cancer in Malmö, 1961–90. Total and percentage cases per age group and average age- and sex-specific incidence per $10^5$ person–years

| *Age (years)* | *Population at risk (average)* | n | *Men (%)* | *Incidence* | n | *Women (%)* | *Incidence* | n | *All (%)* | *Incidence* |
|---|---|---|---|---|---|---|---|---|---|---|
| <34 | | 0 | 0 | 0 | 0 | 0 | 0 | 0 | 0 | 0 |
| 35–39 | 15 335 | 2 | 0.3 | 0.8 | 2 | 0.3 | 0.8 | 4 | 0.3 | 0.8 |
| 40–44 | 15 515 | 6 | 0.9 | 2.6 | 8 | 1.2 | 3.4 | 14 | 1.1 | 3.0 |
| 45–49 | 15 650 | 14 | 2.2 | 6.1 | 5 | 0.8 | 2.1 | 19 | 1.5 | 4.0 |
| 50–54 | 15 974 | 30 | 4.6 | 13.0 | 23 | 3.5 | 9.3 | 53 | 4.0 | 11.1 |
| 55–59 | 15 858 | 43 | 6.6 | 19.1 | 48 | 7.2 | 19.1 | 91 | 7.0 | 19.1 |
| 60–64 | 14 844 | 71 | 10.2 | 34.4 | 58 | 8.8 | 24.0 | 129 | 9.8 | 28.7 |
| 65–69 | 12 945 | 101 | 15.3 | 59.6 | 117 | 17.7 | 53.5 | 218 | 16.6 | 56.1 |
| 70–74 | 10 041 | 101 | 15.3 | 81.5 | 103 | 15.5 | 57.7 | 204 | 15.5 | 67.4 |
| 75–79 | 7 550 | 115 | 17.6 | 135.7 | 121 | 18.3 | 84.9 | 236 | 17.9 | 103.8 |
| >80 | 7 263 | 168 | 25.9 | 247.9 | 178 | 26.5 | 118.6 | 346 | 26.4 | 158.8 |
| All ages | 241 338 | 651 | 100 | 16.7 | 663 | 100 | 17.1 | 1314 | 100 | 18.1 |

**Table II** Pancreatic carcinoma. Estimates of annual change in age-standardised incidence (ASI), city of Malmö, 1961–90

| *Age (years)* | *Annual absolute change of ASI (per $10^5$ person–years)* *Men* | P-*value* | *Women* | P-*value* | *ASI mid-year (1975)* *Men* | *Women* | *Annual change relative to mid-year ASI (%)* *Men* | *Women* |
|---|---|---|---|---|---|---|---|---|
| 35–54 | −0.06 (−0.52 to 0.61)* | 0.61 | 0.11 (−0.01 to 0.24)* | 0.075 | 7.7 | 3.6 | −0.8 | 3.1 |
| 55–64 | −0.11 (−0.80 to 0.58) | 0.75 | 0.48 (0.10 to 0.85) | 0.014 | 32.6 | 18.7 | −0.3 | 2.6 |
| >65 | 0.26 (−1.05 to 1.57) | 0.69 | 1.11 (0.59 to 1.62) | <0.001 | 109.0 | 63.9 | 0.2 | 1.7 |
| All ages | 0.01 (−0.23 to 0.25) | 0.94 | 0.24 (0.14 to 0.35) | <0.001 | 20.0 | 12.1 | 0.0 | 2.0 |

*95% confidence interval.

**Figure 2** Pancreatic carcinoma, age-standardised incidence for women in Malmö, 1961–93. The last 3 years (- - -) are represented by unvalidated cases from the RTR only. ■, 35–54 years; ▲ 55–64 years; ●, >64 years.

whole. This is to a certain extent explained by the higher autopsy rate in Malmö as 20% or more of the new cases reported from Malmö and about 17% of the cases from Sweden as a whole are first detected at autopsy and are clinically unknown cases. The higher incidence in Malmö remains, however, after adjustment for differences in autopsy rates. In Malmö in 1988, for example, the age-adjusted incidence of pancreatic carcinoma, excluding autopsy-detected cases, was 17.7 in men and 12.1 per $10^5$ in women, Corresponding figures for Sweden were 12.0 and 9.1 per $10^5$. (National Board of Health and Welfare, The Cancer Registry, 1991).

The incidence of pancreatic carcinoma in Sweden remained unchanged over the study period in both men and women, while there was a statistically significant increase among women in Malmö. As the autopsy rate in Malmö was fairly steady at around 75% in both men and women, it is not possible to explain the increasing incidence on the basis of a detection bias caused by a change in autopsy rate over time. The number of incidentally detected cases was about 5–6 each year.

The incidence of pancreatic carcinoma is strongly related to age. As the number of old people in the city, especially old women, has grown one could consider age as a confounder. The increasing incidence remained after age adjustment, however.

Our study shows that in order to get a reliable estimate of the incidence one has to use both the Regional Tumour Register and the Cause-of-Death Register. As 20% of the cases found in the RTR only and 40% of the cases found in the CDR only turned out not to be exocrine pancreatic carcinomas it is necessary to validate the carcinomas that have been reported to only one of the two registers. The 81 cases existing in the RTR only or in the CDR only and for which the diagnosis not could be confirmed were evenly distributed during the study period and were found in all age groups.

Even among cases reported to both registers there is a certain proportion of incorrect diagnoses. The estimated 1.8%, based on a 10% random sample of the total 1127 cases existing in both registers, corresponds to 20 cases overall. As we have not validated each of the cases existing in both registers we cannot decide to what extent this degree of misclassification will affect the estimated age- and sex-specific incidence and time trends of disease. We consider, it unlikely, however that the increasing incidence in older women can be explained by an increasing number of incorrect diagnoses in that age group.

The importance of a high and unchanged autopsy rate for a valid estimate of the incidence is emphasised by the fact that not less than 20% of the cases were found incidentally at autopsy.

It is our view that the different incidence in men and women and the increasing incidence over time in older women cannot be explained by a change of the diagnostic validity over time. Neither have we found any results indicating that the increasing incidence can be explained by differences in the reporting rate from the pathologist as 99% of the cases in our survey had been reported to either the RTR or the CDR.

An increasing incidence in older women has also been reported in a study on Olmsted County in Minnesota (Riela *et al.*, 1992). As the changing incidence in our study is most pronounced in women above 64 years it might reflect a cohort effect caused by a growing number of women born 1910 and later being exposed to agents with tumour-promoting or -initiating effects.

The risk of pancreatic cancer is increased in smokers (Cederlöf *et al.*, 1975; Mack *et al.*, 1986; Mills *et al.*, 1988). We have no information on the development of smoking habits among women in Malmö. However, during the 1980s 30% of the women in Malmö were smokers, which means that Malmö belongs to the area in Sweden with the greatest proportion of female smokers (National Central Bureau of Statistics, 1985, 1992; Swedish Institute for Health Services Development, 1991).

Our assumption that at least part of the increasing incidence of pancreatic carcinoma in females in Malmö is explained by smoking is supported by the fact that the age-standardised incidence of pulmonary carcinoma was also higher throughout the period when compared with the national average (National Board of Health and Welfare, The Cancer Registry, 1991). Lung cancer mortality in women in Malmö is at present more than 30% higher than it is in Sweden on average (Swedish Institute for Health Services Development, 1991).

We conclude that in order to obtain a reliable estimate of the incidence of pancreatic cancer it is necessary to use information from more than one register. Differences in the percentage of autopsy-detected, clinically unknown cancers need to be considered as a possible explanation for geographical differences in incidence and time trends of disease occurrence.

In this urban population, in which the autopsy rate remained high, there was a significant increase in the incidence of pancreatic cancer in women older than 55 years, especially older than 64, during the time period 1961–90. Further studies should be undertaken to verify the increased incidence in women and to clarify the role of possible different lifestyle factors, such as smoking and other factors in the environment.

**Acknowledgements**
This study was supported by grants from the Swedish Medical Research Council project no. 17X-8305, the Medical Faculty at Lund University, the Albert Påhlsson and Gunnar Nilsson Foundations and the Foundations for Research at Malmö General Hospital.

### References

BERGE T AND LUNDBERG S. (1977). Cancer in Malmö 1958–1969, an autopsy study. *Acta Pathol. Microbiol. Immunol. Scand.*, sekt. A, suppl. no. **260,** 18–19.

CEDERLÖF R, FRIBERGH L, HRUBEC Z AND LORICH U. (1975). *The Relationship of Smoking and Some Social Covariables to Mortality and Cancer Morbidity in a Probability Sample of 55,000 Swedish Subjects age 18 to 69.* Part 1, pp. 48–49. The Department of Environmental Hygiene, the Karolinska Institute: Stockholm.

DURBIN J AND WATSON GS. (1950). Testing for serial correlation in least-squares regression. *Biometrika*, **37,** 409–428.

McCULLAGH P AND NELDER JA. (1989). *Generalized Linear Models*, 2nd edn., Chapman and Hall: London.

MACK TM, YU MC, HANISCH R AND HENDERSON BE. (1986). Pancreas cancer and smoking, beverage consumption, and past medical history. *J. Natl Cancer Inst.*, **76,** 49–60.

MILLS PK, BEESON WL, ABBEY DE, FRASER GE AND PHILLIPS RL. (1988). Dietary habits and past medical history as related to fatal pancreas cancer risk among adventists. *Cancer*, **61,** 2578–2585.

NATIONAL BOARD OF HEALTH AND WELFARE, THE CANCER REGISTRY. (1991). *Cancer Incidence in Sweden 1988*. pp. 104. National Board of Health and Welfare: Stockholm.

NATIONAL CENTRAL BUREAU OF STATISTICS. (1985). *Living Conditions*. Report no. 42. Health and medical care 1975–1983, (in Swedish). p. 99. National Central Bureau of Statistics: Stockholm.

NATIONAL CENTRAL BUREAU OF STATISTICS. (1992). *Living Conditions*. Report no 76. Health and medical care 1980–1989, (in Swedish). p. 8. National Central Bureau of Statistics: Stockholm.

RIELA A, ZINSMEISTER AR, MELTON LJ, WIELAND LH AND DIMAGNO EP. (1992). Increasing incidence of pancreatic cancer among women in Olmsted County, Minnesota, 1940 through 1988. *Mayo Clin. Proc.*, **67,** 839–845.

SWEDISH INSTITUTE FOR HEALTH SERVICES DEVELOPMENT. (1991) SPRI-REPORT NO.326. *Health and Care on Municipality Level* (in Swedish). pp. 74, 75. Swedish Institute for Health Services Development: Stockholm.

STERNBY NH. (1991). The role of autopsy in cancer registration in Sweden, with particular reference to findings in Malmö. In *Autopsy in Epidemiology and Medical Research*. Riboli E, Delendi M. (eds) pp. 217–222. IARC Scientific Publications no. 112. IARC: Lyon.


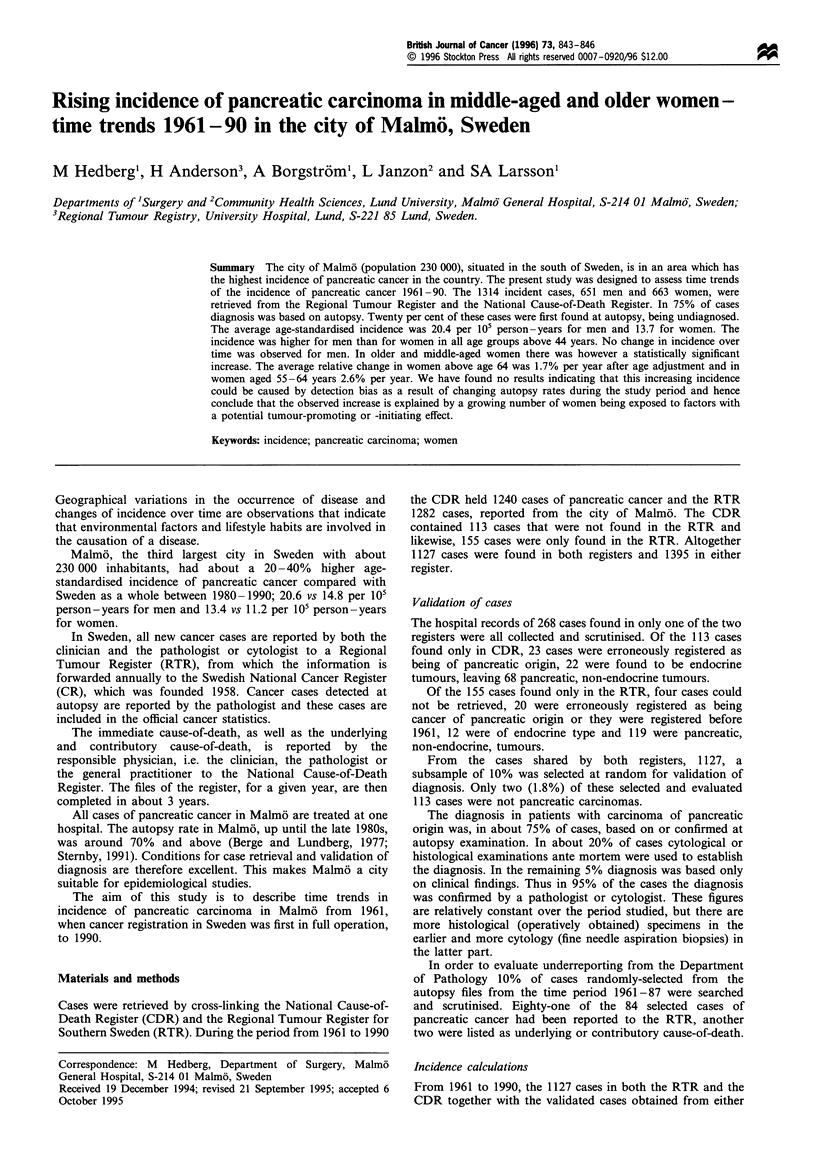

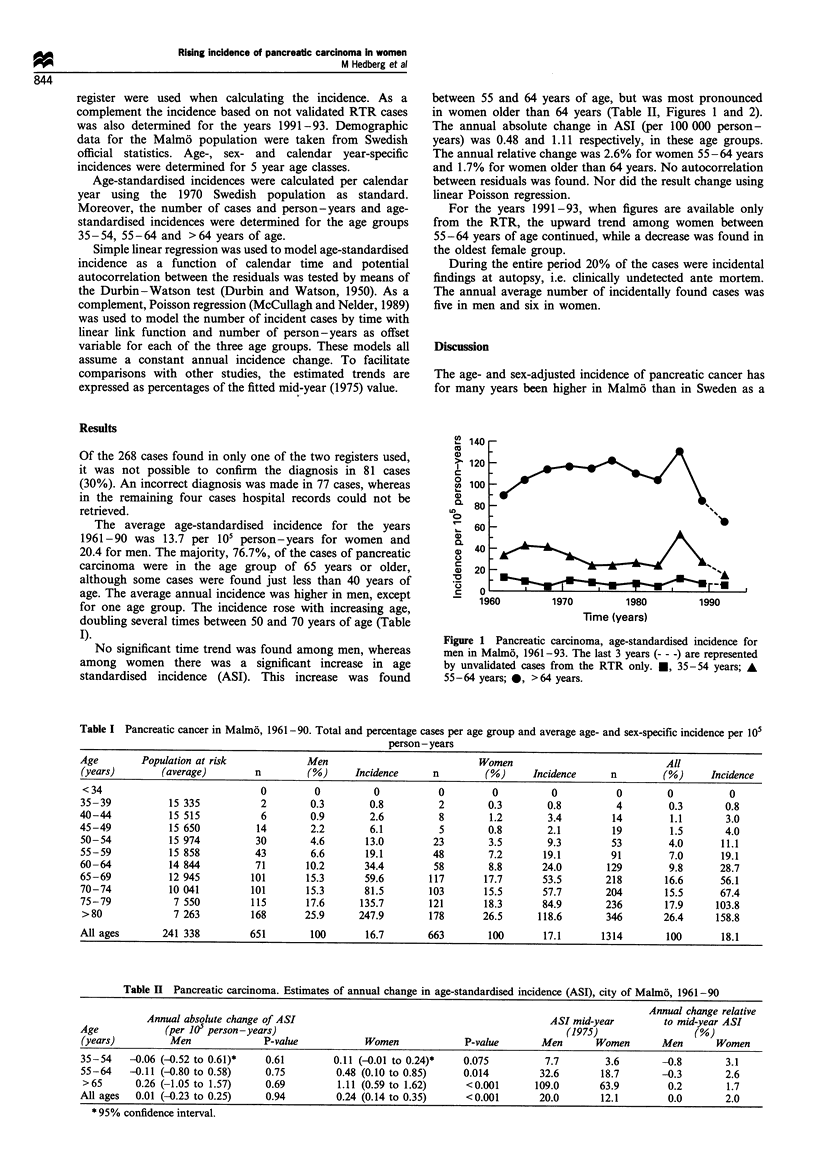

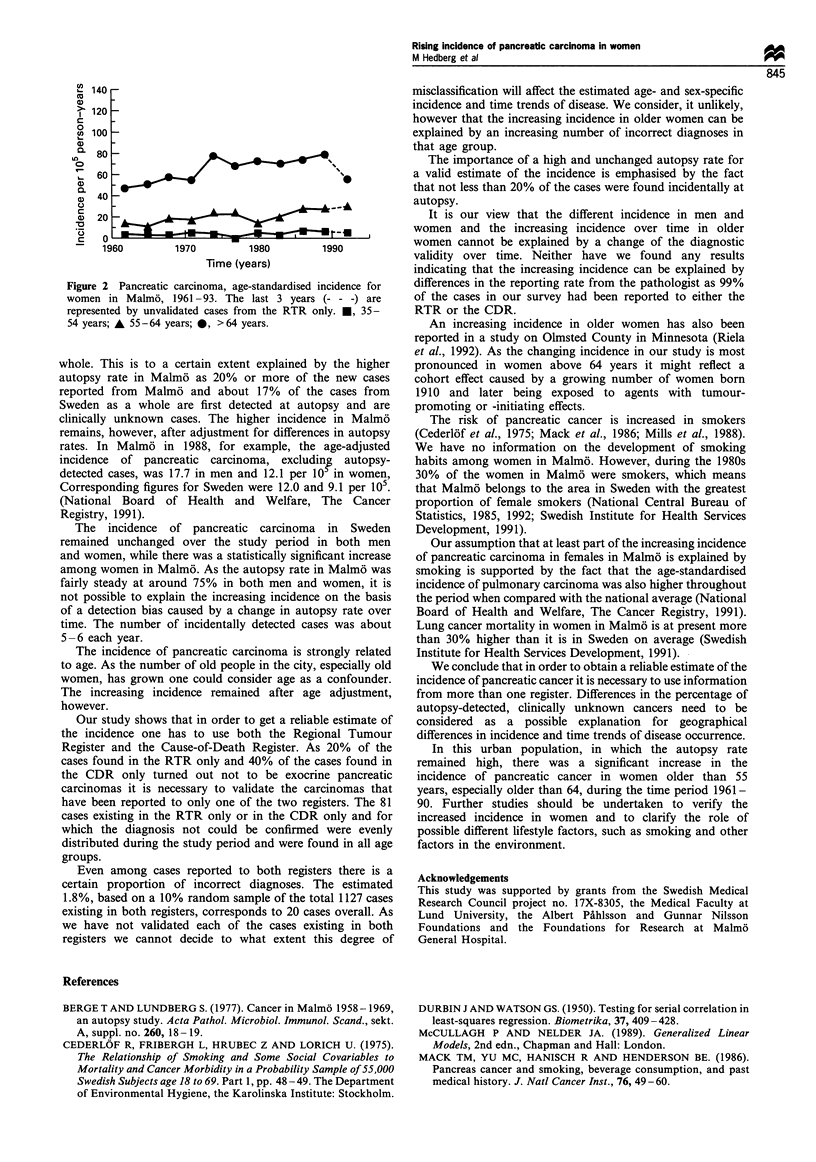

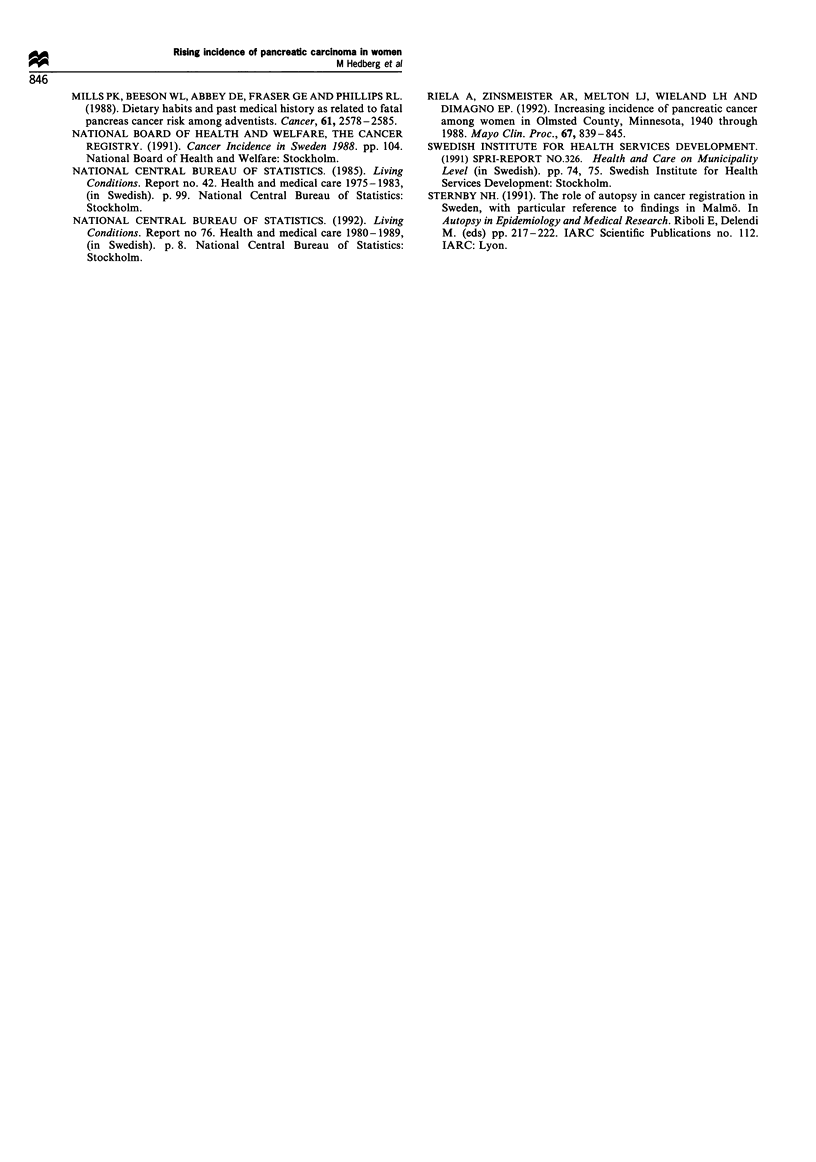


## References

[OCR_00479] DURBIN J., WATSON G. S. (1950). Testing for serial correlation in least squares regression. I.. Biometrika.

[OCR_00485] Mack T. M., Yu M. C., Hanisch R., Henderson B. E. (1986). Pancreas cancer and smoking, beverage consumption, and past medical history.. J Natl Cancer Inst.

[OCR_00494] Mills P. K., Beeson W. L., Abbey D. E., Fraser G. E., Phillips R. L. (1988). Dietary habits and past medical history as related to fatal pancreas cancer risk among Adventists.. Cancer.

[OCR_00519] Riela A., Zinsmeister A. R., Melton L. J., Weiland L. H., DiMagno E. P. (1992). Increasing incidence of pancreatic cancer among women in Olmsted County, Minnesota, 1940 through 1988.. Mayo Clin Proc.

[OCR_00528] Sternby N. H. (1991). The role of autopsy in cancer registration in Sweden, with particular reference to findings in Malmö.. IARC Sci Publ.

